# The sound of swearing: Are there universal patterns in profanity?

**DOI:** 10.3758/s13423-022-02202-0

**Published:** 2022-12-06

**Authors:** Shiri Lev-Ari, Ryan McKay

**Affiliations:** Department of Psychology, Royal Holloway, University of London, Egham Hill, Egham, TW20 0EX UK

**Keywords:** Sound symbolism, Swear words, Profanity

## Abstract

Why do swear words sound the way they do? Swear words are often thought to have sounds that render them especially fit for purpose, facilitating the expression of emotion and attitude. To date, however, there has been no systematic cross-linguistic investigation of phonetic patterns in profanity. In an initial, pilot study we explored statistical regularities in the sounds of swear words across a range of typologically distant languages. The best candidate for a cross-linguistic phonemic pattern in profanity was the absence of approximants (sonorous sounds like *l*, *r*, *w* and *y*). In Study 1, native speakers of various languages (Arabic, Chinese, Finnish, French, German, Spanish; *N* = 215) judged foreign words less likely to be swear words if they contained an approximant. In Study 2 we found that sanitized versions of English swear words – like *darn* instead of *damn* – contain significantly more approximants than the original swear words. Our findings reveal that not all sounds are equally suitable for profanity, and demonstrate that sound symbolism – wherein certain sounds are intrinsically associated with certain meanings – is more pervasive than has previously been appreciated, extending beyond denoting single concepts to serving pragmatic functions.

**Supplementary Information:**

The online version contains supplementary material available at 10.3758/s13423-022-02202-0.

## Introduction

Aficionados of *Star Wars, Star Trek* and *Battlestar Galactica* know that the words “*fierfek*”, “*grozit” and “frak”* are not to be used in polite company. Writers who invent such alien profanity may rely on their intuitions about what makes swear words offensive and transgressive here on earth. The notion that the sounds in such words – their phonemes – contribute to their offensiveness itself transgresses a fundamental linguistic principle: that the connection between the sound and meaning of a word is arbitrary (Hockett, 1959, 1963; de Saussure, 1966/1916).^1^ Nevertheless, a range of authors have suggested that swear words have sounds that render them especially fit for purpose (e.g., Bergen, 2016; Hughes, 2006; Pinker, 2007; Roache, 2016; Vallery & Lemmens, 2021; Wajnryb, 2005). To date, however, there has been no systematic cross-linguistic study of the phonetic patterns in profanity. Here we investigate whether speakers of disparate languages deem certain sounds to be better at expressing profanity than others.

## Sound Symbolism

The general idea that certain phonemes or phoneme combinations are intrinsically associated with certain meanings is known as sound symbolism (D’Onofrio, 2013; Sidhu & Pexman, 2018). For example, across languages the nasal sound *n* is much more likely to occur in words for “nose” than in other words (Blasi et al., 2016; Johansson et al., 2020), and when presented with spiky and curved line drawings, speakers of different languages overwhelmingly favour names such as “takete” and “kiki” for the spiky drawings and “maluma” and “bouba” for the curved ones (Köhler, 1929; Ramachandran & Hubbard, 2001). Sound symbolism can also manifest in a perceived mismatch between a sound and a meaning. Thus, across languages, the sound *m* is statistically unlikely to appear in the word for “skin” (Blasi et al., 2016; Joo, 2020).

Sound symbolic associations are probabilistic, rather than deterministic, in nature. Thus, while the sound *n* is over-represented in words for “nose”, there are many languages in which the word for “nose” does not include it. Similarly, while the sound *m* is under-represented in words for “skin” (Blasi et al., 2016; Joo, 2020), there are languages in which the word for “skin” includes this sound.

Some sound symbolic effects may reflect awareness of reliable co-occurrences in nature. For example, smaller objects tend to produce higher frequencies than larger objects (Coward & Stevens, 2004; Spence, 2011), and adults tend to assign novel words with high formant-frequency vowels, such as *mil*, to small rather than large objects (Sapir, 1929; Thompson & Estes, 2011). Sensitivity to these natural co-occurrences should not rely on linguistic knowledge. Correspondingly, an association between the vowel *i* and the concept “small” occurs persistently across languages from different continents and linguistic lineages (Blasi et al., 2016; replicated in Johansson et al., 2020), and even infants preferentially look at a small rather than a large circle upon hearing vowels with high formant frequencies (Peña et al., 2011).

## Sound Symbolism in Swearing

Several authors have suggested that the main function of sound symbolism is to scaffold language acquisition in childhood (Imai et al., 2008; Kantartzis et al., 2011; Monaghan et al., 2014; Perry et al., 2018; Thompson et al., 2012). But sound symbolism may also facilitate the expression of emotion, attitude or arousal. Humans and nonhuman animals produce harsh, abrasive sounds when distressed and smooth sounds when calm and contented (Nielsen & Rendall, 2011). These tendencies may underpin sound symbolic associations between certain phonemes and profanity (Nielsen & Rendall, 2013).

Numerous authors have offered speculations about specific phonetic patterns in swearing and their potential functions, often suggesting that swear words are rich in plosives (e.g., *p*, *t, k*; Hughes, 2006; Nielsen & Rendall, 2013; Vallery & Lemmens, 2021; Wajnryb, 2005). What is the evidence for such patterns? Van Lancker and Cummings (1999) found that the involuntary swearing of English-speaking patients with Tourette syndrome was phonetically atypical, for example being unusually likely to contain a plosive or fricative consonant (in particular, *f* or *k*) at the beginning of the word. Yardy (2010) compared sounds in English swear words with those in carols and lullabies, reporting that the swear words contained a relatively higher proportion of plosive consonants (e.g., *k*, *t*), while the carols and lullabies contained proportionately more sonorant consonants (e.g., *l*, *w*). Bergen (2016) reported that profane English monosyllables are more likely than control monosyllables to end with a plosive (e.g., *k*). Aryani et al. (2018) found that German speakers rate pseudo-words as more arousing and negative if they include short vowels, voiceless consonants, and – to a degree – plosives. Finally, Reilly et al. (2020) examined predictors of tabooness for both existing English words and novel taboo compounds (e.g., *shitarm*, *doorass*). Words with a higher proportion of plosives (e.g., *k, t*) and affricates (e.g., *ch, j*) were rated as more taboo and considered better candidates for taboo compounding.

## The Present Studies

As reviewed above, there is provisional evidence that English swear words contain a relative preponderance of plosive consonants, and that English natives are sensitive to this pattern. However, previous studies have focused on specific sounds (in particular, plosives) rather than systematically testing for patterns across the full range of phonemic groups. Moreover, it is unknown whether a pattern for plosives – if robust – extends beyond a handful of related Indo-European languages. In some cases, associations between sound and meaning may emerge in a given language due to random co-occurrences of phonological and semantic features. Phonaesthemes are small clusters of words with similar meanings that – initially through coincidence – also have similar phonemic forms, and function as attractors for new words, snowballing into larger language-specific clusters (e.g., in English, 39% of words beginning with *gl*- relate to vision or light, such as *glisten* and *gloaming*; Bergen, 2004). It may be that any association between plosives and profanity is an oddity of English and related languages, attributable to historical and cultural contingency in a particular language rather than to an underlying cognitive bias. In the present studies we explored sound regularities in swearing across several distant languages, combining real-world and experimental data.

In an initial, pilot study we explored whether particular sounds are over-represented or under-represented in the swear words of typologically distant languages. This investigation suggested that the strongest candidate for a cross-linguistic phonemic pattern in profanity was the absence of approximants (sounds like *l*, *r*, *w* and *y*). We thus focused on approximants for our two subsequent studies.

We predicted that individuals would consider words without approximants to be better candidates for swear words, and correspondingly, that one way to sanitize swear words would be to introduce approximants into them. Thus, Study 1 tested experimentally whether native speakers of typologically distant languages are less likely to guess that foreign words are swear words if they contain an approximant versus a control phoneme. Study 2 examined whether, when speakers attempt to reduce the offensiveness of swear words, they do so by introducing approximants. Together the studies investigate whether part of the effectiveness of swear words comes from their sound. In doing so we demonstrate both that sound symbolism is more prevalent than was previously suggested and that the functional role of sound symbolism is broader than has previously been appreciated, extending beyond single concepts to broad pragmatic functions.

## Pilot Study

The goal of our pilot study was to explore whether certain sounds appear less or more frequently in real-world swear words across languages than would be expected by chance, suggesting these sounds are particularly (un)suitable for expressing profanity. We recruited fluent speakers of several typologically distant languages to generate a set of swear words, and compared the frequency of each phonemic group in these swear word sets to the corresponding frequencies in control words. As prior research had suggested that plosives are over-represented in English swear words, we conducted a pre-planned analysis testing plosives’ representation in swear words and exploratory analyses testing the frequency of other phoneme groups against chance. In a follow-up study (see the Online Supplementary Material (OSM) for details) we tested the occurrence of approximants specifically in two further languages.

### Method

#### Data Elicitation

We selected five typologically distant languages: Hebrew, Hindi, Hungarian, Korean and Russian. We recruited 20 fluent speakers of each language via *Prolific* (www.prolific.co). We asked each respondent to provide us with a list of the most vulgar words in their language. Specifically, we requested that they “*...consider both the most vulgar words that are used in [language name] when someone gets hurt or frustrated and the most offensive words that are used to curse someone (i.e., to disparage or insult them).”* Participants were asked to provide a minimum of 5–10 words. Occasionally, participants provided phrases (e.g., the equivalent of “Go suck your sister’s balls”) as well as single words. In total, we collected 86 swear words and phrases in Hebrew, 126 swear words and phrases in Hindi, 68 swear words and phrases in Hungarian, 70 swear words and phrases in Korean, and 94 swear words and phrases in Russian.

As stated in the pre-registration, we only kept words and phrases that were provided by at least two participants. We also consulted native speakers of these languages (except for Hebrew, which the first author speaks as a native language) to identify alternate versions of the same words (e.g., Гандон and Гондон in Russian) and included only the more frequent one of the alternates. Lastly, we excluded racial slurs. At the end of this filtering process, we had 39 swear words and phrases in Hebrew, 19 in Hindi, 27 in Hungarian, 25 in Korean, and 31 in Russian.

To ensure that all the collected words and phrases were indeed profane and to measure their frequency and degree of offensiveness, we presented them to 20 new fluent speakers of each language, again using *Prolific*. To ensure that participants were fluent in the relevant language, they were asked several questions in the survey itself. To be included, participants had to indicate their proficiency as 8 or higher on a 10-point scale and to report that they had used the language in social contexts for at least 1 year after the age of 16. When participants reported low proficiency or no social use, they were replaced with new participants until we reached 20 participants per language group. Each participant was presented with the swear words and phrases in their language in a random order. Participants then rated each swear word or phrase on two dimensions: (1) offensiveness (from “not at all offensive” to “extremely offensive”) and (2) frequency of use (from “extremely rare” to “extremely common in use”). Participants made these ratings by positioning two sliders on 100-point scales. If participants did not consider the word to be a swear word or had not previously encountered it, they could indicate this by pressing a button labeled “This is not a curse word”. We planned on excluding any swear word that was flagged as not a swear word by the majority of participants, but none of the words met this exclusion criterion.

As per our pre-registration we then filtered the words so as to exclude multiple variants of the same word (e.g., *fuck*, *fucking*, *fucker*) to avoid biasing the results. When two words were judged to be variants of the same swear word, we only kept the variant that was rated as more offensive. If the word also appeared in a phrase (e.g., *motherfucker* in *crazy motherfucker*), we only kept the phrase if it included at least one offensive word that did not appear on its own or if it was rated as more common than all its constituent swear words. In those cases, we removed the solo appearance of the constituent words. The final sets therefore included 34 swear words and phrases in Hebrew, 14 in Hindi, 14 in Hungarian, 17 in Korean, and 26 in Russian.

#### Analyses

To test whether certain phonemic groups are over- or under-represented in swear words, the frequency of each phonemic group in the swear words was compared to its frequency in control words. Control words comprised the 100-item Swadesh list of each language, and in the case of Hebrew, the 207-item Swadesh list, as this was also available. The Swadesh list is a set of words for basic concepts compiled by Swadesh (1952). Comparison of its translation into multiple languages allows researchers to trace language lineages. The list has also often been used to address other linguistic questions, from rate of lexical change (e.g., Atkinson et al., 2008; Calude & Pagel, 2011; Pagel et al., 2007; Vejdemo & Hörberg, 2016) to sound symbolism (Blasi et al., 2016). It has been shown that the phonemic inventories in the Swadesh list correlate well with established phonemic inventories, such as the UCLA Phonological Segment Inventory Database (UPSID) (Wichmann et al., 2011). To compare the phonemic distribution of the swear words and control words, we generated 1,000 simulations per language. In each simulation, we randomly sampled from the Swadesh list of the language as many phonemes as were present in the set of swear words for that language.

We then compared the prevalence of each consonant group in the simulated samples to its prevalence in the swear words of that language. To do so, we classified all consonants into plosives, affricates, approximants, sibilant fricatives, non-sibilant fricatives, and nasals. Most of the Swadesh lists we used came from the Automated Similarity Judgment Program (ASJP) database (Wichmann et al., 2022), so we adopted its phonetic coding (Brown et al., 2008). This coding collapses over similar phonemes. Thus:

- Plosives included phonemes coded as ‘b’, ‘d’, ‘g’, ‘p’, ‘t’, and ‘k’. In our set of languages, these cover the phonemes /b/, /d/, /g/, /p/, /t/, and /k/, as well as /pʰ/, /tʰ/, /kʰ/, /bʲ/, /dʲ/, /gʲ/, /pʲ/, /tʲ/, and /kʲ/.
- Affricates included phonemes coded as ‘c’, ‘C’, and ‘j’ in the database. These cover the phonemes /ʦ/, /ʣ/, /ʧ/, and /ʤ/.
- Approximants included phonemes coded as ‘l’, ‘L’, ‘w’, ‘y’, and ‘r’ in the database. These cover the phonemes /l/, /L/, /ʅ/, /ʎ/, /w/, /j/, and “all varieties of “r-sounds” (IPA: r, R, etc.)” (Brown et al., 2008, p.307).
- Sibilant fricatives included phonemes coded as ‘s’, ‘z’, ‘S’, and ‘Z’ in the database. These cover /s/, /z/, /ʃ/, and /ȝ/.
- Non-sibilant fricatives included phonemes coded as ‘f’, ‘v’, ‘8’, ‘x’, ‘h’, and ‘X’ in the database. These cover /f/, /v/, /θ/, /ð/, /x/, /ɣ/, /h/, / ɦ/, /χ/, /ħ/, and /ʕ/.
- Nasals included phonemes coded as ‘m’, ‘n’, ‘N’, and ‘5’ in the database. These cover /m/, /n/, /ŋ/ and /ɲ/.

For each phonemic group in each simulation we calculated the difference between the number of times phonemes from that phonemic group appeared in the swear words versus the control sample. This difference was divided by the total number of phonemes in the swear words for that language, to control for differences in sample size. This measure served as our dependent variable.^2^

**Fig. 1:**
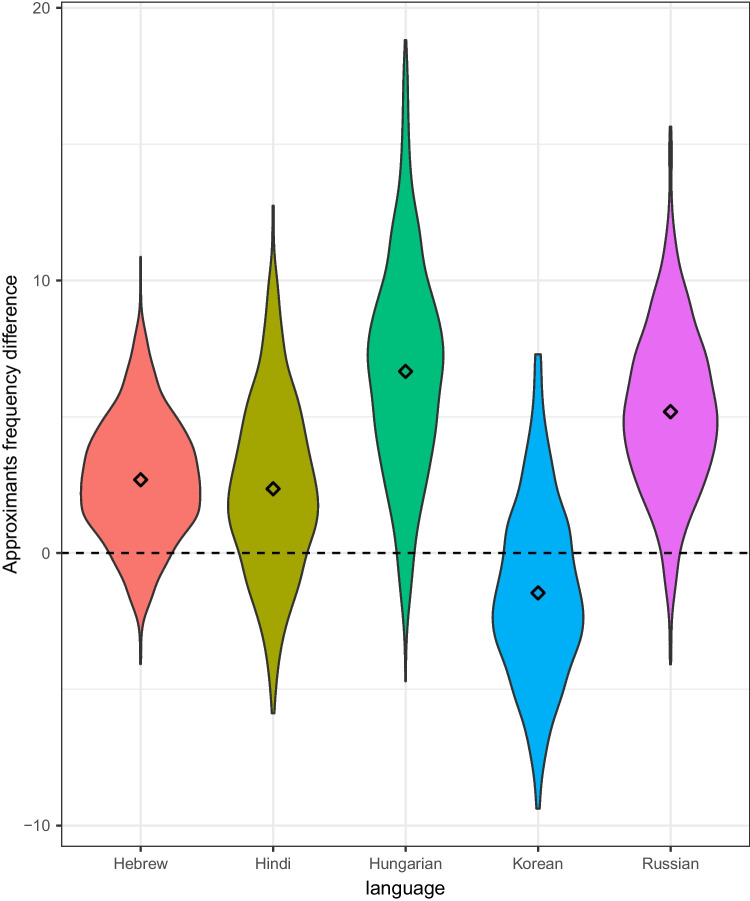
Difference in number of approximants in control word samples versus swear words, by language. Positive values indicate more approximants in control words than in swear words. Diamonds depict per-language averages

We then ran an intercept-only mixed effects analysis for each phonemic group with *Language* as a random variable testing whether the frequency of each phonemic group in the swear words significantly differed from its frequency in the control samples. As only the plosives analysis was motivated by prior claims in the literature, we determined significance using a threshold of *p* = 0.05 for the plosives analysis but applied a Bonferroni correction for the analyses of the other phonemic groups (i.e., setting the *p*-value to 0.01). Results revealed that approximants were under-represented in swear words (β = -3.09, SE = 1.39, *t* = 2.21; see Fig. 1), although this effect was not significant once a Bonferroni correction was applied to the *p*-value. No other phonemic group (including plosives) exhibited higher or lower than expected frequency with or without Bonferroni correction.

## Study 1

The results of our pilot study suggested that swear words are less likely to include approximants than would be expected by chance, implying that approximants are less suitable than other sounds for giving offence. In Study 1 we investigated whether speakers of different languages are sensitive to this sound-symbolic association. We created pairs of pseudo-words, with one member of each pair containing an approximant and the other a control phoneme (an affricate, see below). Participants listened to these pseudo-word pairs and had to guess which member of each pair was a swear word. We hypothesised that the approximant variants would be less likely to be identified as the swear words than the control variants.

### Method

#### Participants

We used *Prolific* (www.prolific.co) to recruit native speakers of each of six languages: Arabic, Chinese, Finnish, French, German and Spanish. We had planned to test native speakers of a new set of typologically distant languages (i.e., distinct from the languages we had included in our pilot study and its follow-up), but decided to add a subsample of French native speakers as in our follow-up to our pilot study (see OSM for details) French had been an exception to the rule that approximants are under-represented in swear words. We reasoned that including French speakers would allow a strong test of the hypothesis of an underlying cognitive bias to associate swear words with a relative dearth of approximants. We aimed to recruit 40 participants per language, but after applying pre-registered exclusion criteria (see below), the final sample comprised 215 participants: 30 Arabic natives (14 female, age: 18–50 years, M = 28.3 years), 37 Chinese natives (24 female, age: 19–47 years, M = 27.3 years), 40 Finnish natives (16 female, age: 19–44 years, M = 31 years), 33 French natives (13 female, age: 18–52 years, M = 29.3 years), 36 German natives (13 female, Age: 19–61 years, M = 29.8 years) and 39 Spanish natives (16 female, age: 19–50 years, M = 26.2 years).

#### Stimuli and Procedure

Participants enrolled in a study entitled *How good is your "sweardar"?* They were told they would hear pairs of words in different languages and that one member of each pair would be a swear word. Their task was to indicate which of the two words they thought was the swear word. Unbeknownst to the participants, the words were in fact pseudo-words, based on existing words in 20 languages (see Table 1).

**Table 1:** Experimental stimuli. All words are presented as they would be rendered in English but they were synthesized using the original orthographies. The replaced phonemes are in boldface

Language	Original word	Approximant version	Affricate version
Albanian	hosto	**w**osto	**j**osto
Albanian	zog	**y**og	**ts**og
Arabic	baida	bai**l**a	bai**j**a
Arabic	insanun	in**w**anun	in**j**anun
Bangla	baba	ba**l**a	ba**ch**a
Bangla	matha	**l**atha	**j**atha
Basque	begi	be**r**i	be**ts**i
Basque	soka	so**l**a	so**ts**a
Catalan	cama	**l**ama	**ch**ama
Catalan	dona	do**y**a	do**ch**a
Chinese	gou	**r**ou	**ch**ou
Chinese	kai	**l**ai	**ch**ai
Czech	dite	di**l**e	di**ch**e
Czech	oko	o**l**o	o**ch**o
German	baum	**l**aum	**ts**aum
German	samen	za**y**en	za**ts**en
Greek	dasos	da**l**os	da**ts**os
Greek	paidi	pe**l**i	pe**ts**i
Hindi	nak	**r**ak	**ch**ak
Hindi	pati	**l**ati	**j**ati
Hungarian	fog	**l**og	**ch**og
Hungarian	mag	**y**ag	**j**ag
Indonesian	ana	a**w**a	a**j**a
Indonesian	hutan	**l**utan	**j**utan
Italian	bastone	**y**astone	**ts**astone
Italian	mano	ma**l**o	ma**ch**o
Japanese	kudamono	**y**udamono	**ts**udamono
Japanese	haku	ha**r**u	ha**ts**u
Korean	sasum	**y**asum	**ch**asum
Korean	tamu	ta**y**u	ta**j**u
Russian	kost	**y**ost	**ts**ost
Russian	spina	spi**l**a	spi**ch**a
Tamil	pen	**w**en	**j**en
Tamil	uppu	u**r**u	u**j**u
Thai	fan	**w**an	**ch**an
Thai	hima	hi**l**a	hi**ch**a
Turkish	kemik	**y**emik	**ch**emik
Turkish	tohu	to**l**u	to**j**u
Vietnamese	bong	**w**ong	**ch**ong
Vietnamese	duong	**y**uong	**ch**uong

Each experimental pair of words was a minimal pair that differed only in that one of the words included an approximant and the other one included an affricate (i.e., ts, ch, or j) in the same position. As per our pilot study, we defined approximants as phonemes coded as ‘l’, ‘L’, ‘w’, ‘y’ and ‘r’ in the ASJP database (Brown et al., 2008; Wichmann et al., 2022), which cover the phonemes /l/, /L/, /ʅ/, /ʎ/, /w/, /j/, and “all varieties of “r-sounds” (IPA: r, R, etc.)” (Brown et al., 2008, p.307). At the same time, we decided to avoid trills when generating words with approximants, as they seem to not belong naturally with the other phonemes, and are indeed not usually defined as approximants (International Phonetic Association, 1999). We contrasted approximants with affricates because our pilot study indicated that affricates were not over- or under-represented in swear words. Therefore, selecting words containing affricates as better swear word candidates would not be due to the suitability of affricates for swear words but to the unsuitability of approximants.

Words were generated by selecting an existing noun that did not include any approximants or affricates (e.g., the word *zog*, meaning “bird”, in Albanian). We then replaced one of its phonemes once with an approximant (e.g., ***y****og*) and once with an affricate (***ts****og*). The replaced phoneme (boldfaced in Table 1) was always a vocalised one, that is, a phoneme followed by a vowel. For each language we generated and presented four pseudo-word pairs: two experimental (affricate vs. approximant, order counterbalanced) and two filler pairs (containing neither approximants nor affricates). Participants were thus presented auditorily with 80 unfamiliar word pairs.

Sound files for the pseudo-words in Albanian, Catalan, Chinese, Czech, Italian, German, Greek, Hindi, Hungarian, Indonesian, Korean, Russian, Tamil, Thai, Turkish and Vietnamese were synthesized using the language-specific speech synthesizers of https://texttospeech.io/text-to-mp3-online. Pseudo-words in Arabic were synthesized using https://ttsmp3.com/text-to-speech/Arabic/, pseudo-words in Bangla with https://www.googletexttospeech.com/p/bangla-text-to-speech-mp3-downloader.html, pseudo-words in Basque with https://www.ehu.eus/seg/ahotss/init.html, and pseudo-words in Japanese with https://www.googletexttospeech.com/p/free-text-to-speech-for-japanese.html. In all cases, we ensured that both words in the pair were generated with the same tone, where relevant, and the same stress pattern.

Trials were presented using *Gorilla* (gorilla.sc/). On each trial the sound played automatically, but participants could choose to replay the audio files up to three times each if they so desired. To give their response on each trial, they moved a cursor to click a box on the left of the screen labelled “First word” or a box on the right labelled “Second word”.

#### Attention Check and Exclusions

In addition to the 80 pseudo-words, we included several pairs of real words in English to serve as attention checks. Each pair comprised a real English swear word paired with a minimal phonetic variant (e.g., *fuck/tuck*, *shit/sit*). Participants who failed to correctly identify the swear word in more than 25% of these trials were excluded (*n* = 18). We also eliminated any participant who responded before the end of the second audio file on 10% or more trials (*n* = 6). We had planned to exclude any participant who took more than three times the median response time (RT) to respond (without having replayed either of the audio files) on 10% or more trials, but the only participant who met this criterion had already been excluded. In addition, we excluded any trial where the participant responded before the second audio file had finished playing (0.3% of all trials), or where the participant did not replay the audio files yet responded at an RT that was more than three times the median RT for trials where the audio files were not replayed (1.2% of all trials).

Our pre-registration stipulated one final exclusion criterion: that we would remove any participant who made 10 (or more) of the same response in a row. We had reasoned that such behaviour would reflect a lack of proper engagement with the task, but on reflection we realized that runs of 10 or more could occur quite easily by chance in a sequence of 88 trials (such that our criterion would exclude 7.58% of attentive participants on average). We therefore opted not to exclude such participants in our main analysis, but we report an additional analysis with these exclusions in a footnote (yielding virtually identical results; see footnote 3).

### Results

To test whether participants were less likely to select the words with approximants as the swear words, we coded selection of the word with an affricate as 1 and selection of the word with an approximant as 0. We then used the lme4 package (Bates et al., 2010) in R (R Core Team, 2020) to run an intercept-only logistic mixed effects model on swear word selection with *Participants* and *Items* as random variables. As predicted, this analysis revealed that participants were significantly less likely to judge that the words with approximants were swear words (β = 0.52, SE = 0.08, *z* = 6.76, *p* < .001). This is equal to selecting the affricates on 63% of trials.^3^

**Fig. 2:**
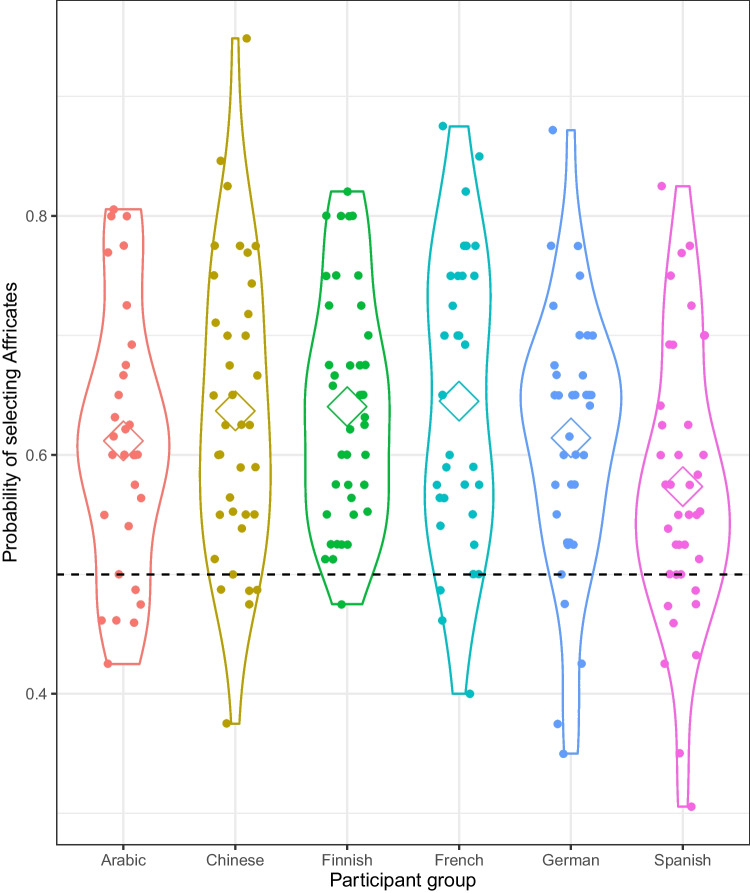
Proportion of trials in which participants (grouped by native language) identified the word with the affricate rather than the approximant as the swear word. Diamonds depict group averages, and the dashed line denotes chance. Plot generated using the tidyverse package (Wickham et al., 2019)

Exploratory analyses indicated that this pattern held across all participant groups (see Appendix A Tables 2, 3, 4, 5, 6, 7 and 8 and Fig. 2). Notably, French speakers, whose native language does not align with the cross-linguistic pattern but instead is rich with swear words that include approximants (see OSM), also selected the affricates over the approximants on around 63% of the trials. This suggests that the task taps underlying cognitive biases rather than reflecting specific linguistic knowledge.

The results provide strong evidence that participants are less likely to select words with approximants than words with affricates when asked to detect the swear word (to ensure that this result was not due to participants identifying which stimulus was most *word*-like, we ran a follow-up experiment – see OSM for details).

## Study 2

Study 1 showed experimentally that individuals judge words containing approximants as less likely to be swear words, implying that approximants are less suitable than other sounds for giving offence. Here we tested this idea using a different type of real-world data: minced oaths (Hughes, 2006; McCord, 1968). Minced oaths are sanitized versions of swear words formed by altering one or more sounds in the original word (e.g., transforming *damn* to *darn*). If approximants are perceived as particularly inoffensive, one might expect that when altering a swear word’s sounds so as to render it more suitable for polite company, speakers will be particularly likely to introduce an approximant. We thus hypothesized that minced oaths would contain more approximants than the swear words they were derived from.

### Method

#### Data Collection

To test our hypothesis, we collected all words in the Oxford English Dictionary (OED, n.d.) whose definition included the words *euphemism*, *euphemistic, minced* or *mincing* together with *alteration*. We complemented this list with additional examples that were listed in the Wikipedia entry on Minced Oaths (“Minced oath”, 2021). As we were only interested in swear words whose alteration is intended to decrease offensiveness, we excluded references to God or Christ, as alterations in those cases are intended to avoid saying God’s name. Additionally, we ignored Cockney rhyming slang. Lastly, we excluded cases where the minced oath was only the first letter of the original word, as no sound substitution had taken place in these instances. Our dataset included 67 minced oaths that were altered versions of 24 swear words, as some swear words had many minced versions (e.g., *fucking* has many altered versions including *frigging* and *effing*). While the set is relatively small, it includes all altered swear words in English attested by the OED (*N* = 43) and all the additional minced oaths that were listed in the Wikipedia entry on Minced Oaths (*N* = 24).

### Results

For each minced oath, we counted how many approximants it included as well as how many approximants the original version had (e.g., *frigging* – 1, *fucking* – 0). We used British rather than American pronunciation, for example “darn” was scored as having no approximants. Because the approximant counts are discrete, the data did not meet the assumptions of a paired t-test. We therefore conducted a Wilcoxon signed rank test with the Pratt method for incorporating differences of zero, using the scipy package (Virtanen et al., 2020) in Python 3. The results showed that approximants were more frequent in minced oaths than in the original swear words (29 vs. 12; *W* = 58, *p* < .001), indicating that when speakers altered swear words to render them less offensive, they did so by introducing approximants. In the OSM we demonstrate that this result is robust irrespective of sample selection and coding choices.

## General Discussion

*Trying to express anger using a swear word full of gentle, soft sounds... would be the verbal equivalent of angrily trying to slam a door fitted with a compressed air hinge.*

~ Rebecca Roache (2016)

Swear words have a unique linguistic power. Swearing in public is illegal in many countries and profanity is a major target for censorship in the arts and entertainment industries (Bergen, 2016). Swearing elicits physiological responses such as elevated heart rate and increased galvanic skin response (Bowers & Pleydell-Pearce, 2011; Buchanan et al., 2006; Harris et al., 2003). Moreover, swearing aloud increases tolerance to pain (Stephens et al., 2009; Stephens & Robertson, 2020; Stephens & Umland, 2011) and boosts physical performance (Stephens et al., 2018).

What gives swear words their potency? Part of the answer, of course, may lie in what these words literally refer to: after all, the usual suspects include taboo topics such as excretion and sex. However, the sounds in swear words may also play an important role.

Our findings indicate that not all sounds are equally suitable for profanity. In an initial, pilot study we explored statistical regularities in the sounds of swear words across a set of typologically distant languages. The most promising candidate for a universal phonemic pattern in profanity to emerge from this analysis was the absence of approximants (sonorous sounds like *l*, *r*, *w* and *y*). Study 1 confirmed that native speakers of various typologically distant languages were relatively unlikely to identify words containing an approximant as swear words. It may be that approximants are sound-symbolically associated with calm and contentment, and so are unsuitable for giving offence (Nielsen & Rendall, 2011, 2013; Yardy, 2010). In Study 2 we found that sanitized versions of swear words – minced oaths – contain significantly more of these sounds than the swear words they were derived from. According to Hazen (2020), minced oaths allow “for restrained fist-shaking at the universe”. Our findings suggest that approximants are a relevant “restraint” – the verbal equivalent of fitting a compressed air hinge to a door (Roache, 2016).

Though we focused primarily on approximants, future studies may identify other phonemic groups particularly suitable or unsuitable for profanity. For example, studies with larger samples from more languages might allow other, weaker, effects to emerge. Alternatively, it may be that some phonemic effects occur only in certain word positions (e.g., stressed syllable, word ending, vocalized position) or only in combination with other phonemes. For example, Bergen (2016) reported that monosyllabic nonwords sound more profane to native English speakers when they end in a consonant than a vowel.

Our findings demonstrate that sound symbolism is more pervasive, with a broader functional role, than has previously been appreciated, extending beyond single concepts (such as object size) to broad pragmatic functions. This has both practical and theoretical implications. At the practical level, using words rich in approximants may help defuse tense social situations and so may be important in a range of real-world contexts (e.g., relationship conflict, diplomacy, hostage negotiation). At the theoretical level, our results suggest a functional role for sound symbolism that extends beyond supporting language acquisition in childhood (Imai et al., 2008; Kantartzis et al., 2011; Monaghan et al., 2014; Perry et al., 2018; Thompson et al., 2012). Specifically, our findings suggest that sound symbolism can facilitate the pragmatic expression of emotion, attitude or arousal (Nielsen & Rendall, 2011, 2013). Other fields of linguistics, such as semantics and historical linguistics, may benefit from considering how sounds can be modified to better exploit other pragmatic functions. While Study 2 focussed on minced oaths and offensiveness, word alterations in other vocabulary domains may reflect other sound symbolic patterns and serve other pragmatic functions (e.g., cajoling, appeasing, expressing authority).

We acknowledge three caveats. First, while our results demonstrate clearly that speakers of a range of languages tend to judge that words with approximants are not good candidates to be swear words, this finding is about *perceptions* of swearing, rather than swearing itself. Nevertheless, our experimental approach is consistent with a long tradition in sound symbolism research. For instance, it has long been accepted that there is a sound symbolic association between high front vowels such as *i* and small size, yet for decades this consensus relied solely on experiments (e.g., Newman, 1933; Peña et al., 2011; Sapir, 1929; Tarte & Barritt, 1971; Thompson & Estes, 2011). It was only very recently that an association between the vowel *i* and the concept “small” was demonstrated to be a statistical regularity across actual human languages (Blasi et al., 2016).

Second, we do not mean to suggest that the presence of approximants is sufficient to render words inoffensive: again, our findings are probabilistic rather than deterministic. What our results point to is an underlying cognitive bias, a predisposition that will have acted in concert with historical accident to shape the evolution of swear words. Just as the association between nasal sounds and words for “nose” does not manifest in every language – or even in *most* languages (Blasi et al., 2016) – we should not expect that the pattern we have identified will manifest in every language, and even languages that reflect the pattern are likely to have swear words with approximants, though fewer than would be predicted by their sound system.

Third, although we recruited speakers of different languages for Study 1, they were all familiar with English. We cannot exclude, therefore, the possibility that their performance in the “sweardar” experiment reflected their familiarity with phonetic patterns in English. We think this unlikely, as native French speakers, whose language does not exhibit this pattern, demonstrated numerically the strongest effect in the experiment. This suggests that performance in the experiment does not simply mirror linguistic knowledge. Moreover, our pilot study results indicated that approximants were at least as under-represented in the swear words of some of the other languages we investigated (e.g., Hungarian, Russian) as they were in English.

So, are swear words “universally patterned on the basis of sound” (Wajnryb, 2005, p. 205)? Our results point to a robust cross-linguistic sound symbolic association in the minds of human speakers. As to the wider universe, the jury is out: surprisingly, according to *The Hitchhiker’s Guide to the Galaxy,* the rudest word in the universe is “Belgium”, which contains an approximant.^4^

### Supplementary Information

ESM 1 (DOCX 167 kb)

## Data Availability

Our hypothesis, method and analyses for our pilot study and Study 1 were pre-registered with AsPredicted.com (Pilot study, #38283: https://aspredicted.org/Z1P_KCW; Study 1, #60913: https://aspredicted.org/blind.php?x=/V51_7J4). Our data, analysis scripts and materials for all three studies are available on the Open Science Framework at https://osf.io/fp92q/?view_only=622565ff35ed454fad386c42c10106b0

## Appendix A – Tables of results for Study 1

**Table 2:** Results of main analysis

Random effects				
Group name	Variance	STDEV		
Participant (Intercept)	0.1550	0.3937		
Audio (intercept)	0.1908	0.4368		
Fixed effects				
	*β*	SE	z value	p-value
(intercept)	0.52217	0.07771	6.719	1.82e-11

**Table 3:** Results for Arabic speakers

Random effects				
Group name	Variance	STDEV		
Participant (Intercept)	0.1213	0.43483		
Audio (intercept)	0.1956	0.4422		
Fixed effects				
	*β*	SE	z value	p-value
(intercept)	0.4889	0.1133	4.314	1.6e-05

**Table 4:** Results for Chinese speakers

Random effects				
Group name	Variance	STDEV		
Participant (Intercept)	0.2392	0.4891		
Audio (intercept)	0.2210	0.4701		
Fixed effects				
	*β*	SE	z value	p-value
(intercept)	0.6212	0.1238	5.016	5.28e-07

**Table 5:** Results for Finnish speakers

Random effects				
Group name	Variance	STDEV		
Participant (Intercept)	0.08228	0.2868		
Audio (intercept)	0.33821	0.5816		
Fixed effects				
	*β*	SE	z value	p-value
(intercept)	0.6241	0.1166	5.353	8.66e-08

**Table 6:** Results for French speakers

Random effects				
Group name	Variance	STDEV		
Participant (Intercept)	0.1948	0.4413		
Audio (intercept)	0.1549	0.3936		
Fixed effects				
	*β*	SE	z value	p-value
(intercept)	0.6473	0.1162	5.569	2.55e-08

**Table 7:** Results for German speakers

Random effects				
Group name	Variance	STDEV		
Participant (Intercept)	0.1358	0.3685		
Audio (intercept)	0.3185	0.5643		
Fixed effects				
	*β*	SE	z value	p-value
(intercept)	0.5070	0.1227	5.353	3.58e-05

**Table 8:** Results for Spanish speakers

Random effects				
Group name	Variance	STDEV		
Participant (Intercept)	0.1320	0.36634		
Audio (intercept)	0.1575	0.53969		
Fixed effects				
	*β*	SE	z value	p-value
(intercept)	0.3201	0.1010	3.169	0.00153

## Footnotes

1 It is worth noting that many thinkers arguing for arbitrariness as a fundamental aspect of language, such as Hockett, were active after Sapir and Köhler’s findings on sound symbolism were already well known. Their argument was not necessarily that sound symbolism is completely absent in language but that its presence is limited to a few words here and there, and that it does not play a meaningful role in communication. Thus, Hockett writes: “Human language is *almost* wholly arbitrary” (Hockett, 1959, p. 34; emphasis added).

2 This analysis differs from the one we pre-registered because once we carried out the pre-registered analysis, we realised it was biased in that it led to some languages receiving higher weight than others. See the Online Supplementary Material (OSM) for the pre-registered analysis. The main result reported here – under-representation of approximants - is significant in the pre-registered analysis.

3 We then repeated the analysis after removing 31 participants who provided the same response 10 or more times in a row (as per our pre-registration). Participants were again less likely to select the words with approximants as swear words (β = 0.53, SE = 0.08, *z* = 6.90, *p* < .001).

4 We can’t resist noting that, in reality, the Belgium case accords with our thesis, because in this context “Belgium” is actually a minced oath – a substitute for “fuck” (Inglis-Arkell, 2012). Douglas Adams’ American publishers objected to the word “fuck” in the original text of *Life, the Universe, and Everything* (the third book in the *Hitchhiker's Guide to the Galaxy* series), and Adams responded by replacing it with “Belgium” and adding some embellishments about the word “Belgium” being forbidden everywhere but Earth (where people were too stupid to know what it meant). The joke plays on the stereotype of Belgium as bland and inoffensive (Mason, 2022), but the approximant in the word might have helped seal the deal.
