# Supplementary material for: The sound of swearing: Are there universal patterns in profanity?
Source: Psychon Bull Rev. 2022 Dec 6;30(3):1103–14. doi: 10.3758/s13423-022-02202-0 (PMC10264537; doi:10.3758/s13423-022-02202-0)
Supplement: Supplementary file 1 — (DOCX 167 kb) [file 13423_2022_2202_MOESM1_ESM.docx]

**The Sound of Swearing: Supplementary Material**

**Pilot: Pre-registered analyses**

The analysis reported below is that which we pre-registered. Once we conducted it, however, it became clear that the analysis is biased in that the weight of each language in it is proportional to the number of swear words we elicited for that language. For example, Hebrew has twice the weight of Hindi in this analysis because we elicited twice as many swear words in Hebrew as we did in Hindi. In the manuscript proper we report a modified analysis that corrects for this bias. Still, as the uncorrected analysis is the analysis that we pre-registered, we report it here. As can be seen below, the under-representation of approximants we focus on in the paper is significant in this uncorrected analysis.

To compare the phonemic distribution of the swear words and control words, we generated 1000 simulations. In each simulation, we randomly sampled from the Swadesh lists of all five languages such that for each language we sampled as many phonemes as were present in the set of swear words for that language. For the test of plosive over-representation, we examined in how many simulations plosives were at least as frequent as in the swear word set. If this occurred in fewer than 50 simulations out of 1000, then plosives are overrepresented in swear words at a *p*-level of 0.05.

We also compared the representation of the other consonant groups, namely affricates, approximants, sibilant fricatives, non-sibilant fricatives, and nasals. As these were exploratory analyses, we defined as significant a swear word frequency for the relevant consonant group that was above or below 99% of the control simulations.

The results of the pre-registered analysis showed plosives to be over-represented in swear words (132 vs M=114, 95^th^ percentile=129). The analysis also revealed that non-sibilant fricatives and approximants are under-represented in swear words (Approximants: 66 vs M=86, 1^st^ percentile=68; Non-sibilant fricatives: 36 vs M=52, 1^st^ percentile=38).

**Pilot: Voicing analysis**

The cross-linguistic analyses suggested that approximants are the best candidate for a sound symbolic association in swear words. As all approximants are voiced, however, one might wonder whether it is approximants that are under-represented in swear words or voiced phonemes that are under-represented in swear words. To test this, we repeated the simulations and corrected analyses reported in the paper, only this time we did not code the phonemic group of each phoneme but rather coded whether or not it is voiced.

The following phonemes were coded as voiced: /b/, /d/, /g/, /z/, /ȝ/, /v/, /l/, /L/, /ʅ/, /ʎ/, /w/, /j/, “all varieties of “r-sounds”, /m/, /n/, /ŋ/, /ɲ/, and /ʤ/. The following phonemes were coded as voiceless: /p/, /t/, /k/, /s/, /ʃ/, /f/, and /ʧ/. Phonemes coded in the ASJP database as '8', 'h', 'X', ‘x’, and 'c' were excluded as these notations collapse over voiced and voiceless phonemes. We then conducted simulations similar to the corrected ones conducted for all other phonemic groups. That is, we conducted 1,000 simulations per language such that in each simulation we randomly sampled as many phonemes from the language’s Swadesh list as there were in the set of swear words for that language. Then we compared the prevalence of voiced phonemes and of voiceless phonemes in each simulated sample with their prevalence in the swear words and normalized the values by dividing them by the sample size for that language. These were the dependent measures of our analyses. We then ran intercept-only mixed effects analyses, once for voiced phonemes and once for voiceless phonemes, with Language as a random variable. The analyses tested whether the frequency of voiced and voiceless phonemes in the swear words significantly differed from their frequency in the control samples. Neither analysis reached significance (voiced: β=-0.3664, SE= 2.1104, t=-0.174; voiceless: β=-0.2987, SE=1.7941, t=-0.166). Therefore, even though approximants are voiced, voicing did not drive the under-representation of approximants in swear words.

**Pilot: Additional samples**

Following the initial promising results regarding approximants, we decided to test the occurrence of approximants in two more languages, English and French. This additional study was conducted in precisely the same manner as the studies with the five initial languages, except that our only prediction, and therefore only planned test, regarded the frequency of approximants: we predicted that approximants would be under-represented in swear words relative to control words. The study was pre-registered with AsPredicted.com (#48696: <https://aspredicted.org/VNC_TJZ>).

In total, we collected 30 swear words and phrases in English and 32 in French. After filtering, we had 28 swear words and phrases in English and 22 in French. Results revealed that approximants were under-represented in English (11 vs M=16.9, 5^th^ percentile=11) though not in French (20 vs M=14.7). The results for French might seem surprising, but this is partly because the phoneme frequency distribution in the French Swadesh list (Swadesh, 1952) differs markedly from other estimates of phonemic frequencies in French (Malécot, 1974). While in the French Swadesh list approximants comprised only 11% of all phonemes, they comprised 15.5% of all phonemes in Malécot (1974), slightly above their frequency in French swear words (15.3%).

It is worth noting that the discrepancy between phoneme frequencies in the French Swadesh list and in the French language as a whole is specific to French. When examining estimates of phoneme frequencies for the other languages in our sample – where those could be obtained – the values tended to be quite similar to those in the Swadesh list: 12.1% vs 12.5% for Hebrew (Silber-Varod et al., 2017), 11.8% vs 11.0% in Korean (Shin, 2010). When they differed, the differences were small, could go in either direction (e.g., higher frequency than in the Swadesh list for English, but lower frequency than in the Swadesh list for Hindi), and crucially, in all cases, they aligned with the direction of the effect we found in our analysis. That is, even when the estimates of the frequency of approximants in the language differed from their frequency in the Swadesh list, the estimates remained higher than the frequency in swear words, except for Korean, where according to both the Swadesh list and published estimates, approximants are not less common in swear words than in the language in general.

Prior literature has suggested that plosives are over-represented in swear words. We did not find support for this claim in the corrected analyses reported above for our initial five languages. That said, as most of the prior suggestive findings in the literature regarded English, we decided to run an additional exploratory test to examine whether plosives are over-represented in English. Results indicated that this is the case (41 vs M=29, 95^th^ percentile=37).

**Study 1: “Word-dar” control experiment**

Our main “sweardar” experiment (Study 1) demonstrated that participants are less likely to consider a pseudo-word to be a swear word if it contains an approximant. One alternative interpretation of these results could be that participants did not try to identify the swear word but instead tried to identify which of the two stimuli was a word. In other words, it is possible that approximant variants simply seem less word-like, in general, than affricate variants, and not just less likely to be swear words. This seems unlikely given that our instructions made it clear that both stimuli on each trial were words. Nevertheless, we conducted a control experiment to fully rule out this possibility empirically. To do so, we repeated our sweardar experiment, only this time, we asked participants to guess which of the two stimuli was a word (rather than a swear word). We predicted that, unlike in the swear-dar experiment, in this case participants would respond at chance.

***Participants***

We used Prolific ([www.prolific.co](http://www.prolific.co)) to recruit 40 speakers of Chinese (26 females; Age: 19-58, M=32.8) who did not participate in the prior “sweardar” experiment. We replaced one of the participants as, when asked to list the languages they spoke, the participant only listed English and Vietnamese.

***Stimuli and Procedure***

The stimuli and procedure were identical to those in the original sweardar experiment with the following exceptions: (1) Instead of telling participants that they would hear pairs of words and that their task was to indicate in each case which of the two words was a swear word, participants were told that they would listen to pairs of *recordings*, and that only one recording in each pair was a recording of a *word*. Their task was to indicate which of the two recordings was of the real word. (2) The attention checks were replaced. In line with the new framing and instructions, only one recording in each pair constituted a real English word and it was not a swear word. The pairs were: cat/nat, desk/fesk, food/sood, and house/shouse.

***Results***

Our initial analysis followed our prior exclusion criteria exactly. This entailed excluding 2 participants who failed more than 25% of the attention check trials, and 15 participants who took more than three times the median RT to respond (without having replayed either of the audio files) on 10% or more trials; thus, leaving *N*=23 participants. An intercept-only logistic mixed effects model on word selection with *Participants* and *Items* as random variables revealed that participants were no less likely to judge that the recordings with approximants were words than the recordings with affricates (β=0.03, SE=0.13, *z*=0.22, *p*=.829). This is equal to selecting the affricates on 50% of trials.

To ensure that the results were not influenced by the exclusion of participants, we re-ran this analysis in two ways: first, ignoring our criteria about overly slow responses altogether; and second, excluding responses that were more than 3x the median RT but keeping the non-slow responses from participants who responded slowly on 10% or more trials. In both cases *N*=38, and in both cases the effect is still null (*p* = .984 and *p*= .790, respectively, indicating that pseudo-words with affricates were selected on 50% and 51% of trials, respectively). As for why participants were more likely to have longer response times, we suspect this may be because the task was harder as participants had no intuition or biases to rely on. In the sweardar task, participants could follow their bias that approximants are less appropriate in swear words, so responded more quickly. Here, responses were at random, as there was no guiding bias.

Finally, we ran a further analysis to compare the performance of the Chinese group in this control experiment with that of the Chinese group in the original sweardar experiment. The difference between the two groups is highly significant (β=-0.60, SE=0.12, *z*=-5.18, *p*<.001; see Figure S1).

**
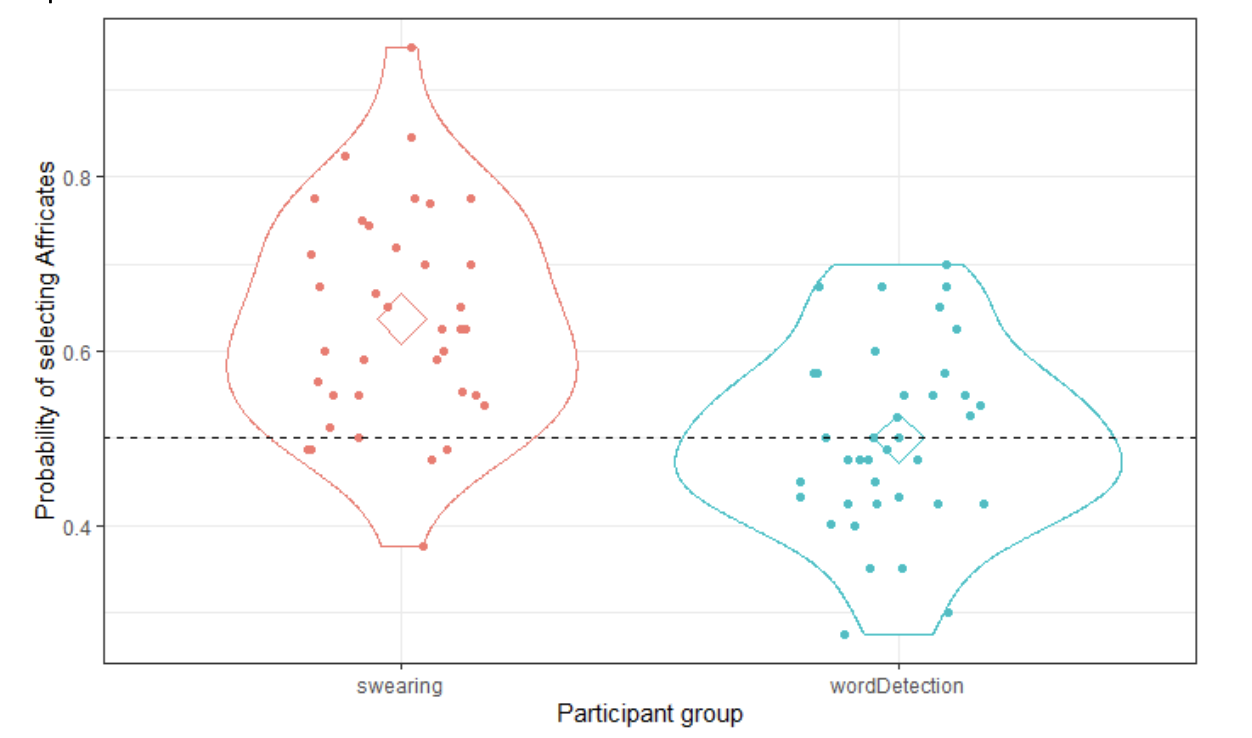
Figure S1.** Selection of the variants with affricates across the two groups of Chinese speakers. The horizontal line indicates chance performance. Every dot represents the average of one participant.

**Study 2: Robustness checks**

As with any analysis, the analysis of Study 2 requires making some choices. To ensure our results were not influenced by any of the choices we made, we carried out several robustness checks.

First, we repeated the main analysis using American rather than British pronunciation, e.g., scoring “darn” as having one approximant. These results revealed the same pattern: approximants were more frequent in minced oaths than in the original swear words, *W*=107, *p*<.001.

Second, our dataset included minced oaths from both the OED and Wikipedia as we wanted the dataset to be comprehensive. That said, Wikipedia might be considered a less reliable source as articles can be modified by anyone. To ensure that our results were not swayed by the inclusion of this source, we conducted an additional analysis that included only the items from the OED (*N*=43). The results of this analysis revealed the same pattern: more approximants in minced oaths than in the original swear words (*W*=37, *p*=.004).

Third, many swear words have several altered versions (e.g., *frigging* and *effing*). While it seems reasonable to assume that each alteration is independent of the others, one might worry that our results are driven by alterations to only a small proportion of the swear words – those that have many altered versions. As an additional robustness check, we carried out an analysis in which each swear word from the OED and Wikipedia appeared only once. If the swear word had multiple altered versions, we included the average number of approximants across its altered versions. This analysis also yielded the same results, i.e., a higher number of approximants in the minced oaths than in the original swear words they were derived from (*W*=21.5, *p*=.009).

**Study 2: The prevalence of plosives in minced oaths**

Our studies focus on the absence of approximants as a cross-linguistic phonemic pattern in profanity. However, most prior research had suggested that plosives are particularly suitable for giving offence. While we did not find cross-linguistic evidence for that proposal in our pilot study, the results for English suggested that there might be an English-specific tendency to include plosives in swear words, which might explain prior suggestions, as they were all proposed by English speakers or based on analyses of English or German. As the set of minced oaths was in English, we decided to run an exploratory analysis to test whether minced oaths consist of fewer plosives than the original swear words, as would be expected if plosives are particularly potent for giving offence. The results of the Wilcoxon signed rank test did not support this hypothesis (*W*=592, *p*=.217). Plosives were equally likely to appear in the altered versions as they were in the original swear word. For example, a change of *fuck* to *flip* only replaces one plosive (*k*) with another one (*p*).

**References**

Malécot, A. (1974). Frequency of occurrence of French phonemes and consonant clusters. *Phonetica, 29, 3*, 158-170.

Shin, J. (2010). Phoneme and syllable frequencies based on the analysis of entries in the Korean dictionary. *Communication Sciences & Disorders, 15, 1*, 94-106.

Silber-Varod, V., Latin, M. & Moyal, A. (2017). Frequency of Hebrew phonemes and phoneme clusters in a data-driven approach. (in Hebrew). *Literacy and Language (Oryanut Ve-Safa), 6*, 22–36.

Swadesh, M. (1952). Lexico-statistic dating of prehistoric ethnic contacts: with special reference to North American Indians and Eskimos. *Proceedings of the American Philosophical Society, 96, 4*, 452-463.
